# Supplementary material for: Novel Nitric Oxide Donor Dinitroazetidine-Coumarin Hybrids as Potent Anti-Intrahepatic Cholangiocarcinoma Agents
Source: Molecules. 2022 Jun 22;27(13):4021. doi: 10.3390/molecules27134021 (PMC9268168; doi:10.3390/molecules27134021)
Supplement: Supplementary file 1 [file molecules-27-04021-s001.zip › molecules-1730396-supplementary.pdf]

Supplementary Information

Novel Nitric Oxide Donor Dinitroazetidine-Coumarin Hybrids as Potent Anti-Intrahepatic Cholangiocarcinoma Agents

Zhihui Yu [1,†], Mengru Li [2,†], Shiqi Guo [1], Weijie Wang [1], Feng Qu [1], Yulei Ma [2], Hongrui Liu [2,*] and Ying Chen [1,*]

[1] Department of Medicinal Chemistry, School of Pharmacy, Fudan University, Shanghai 201203, China

[2] Department of Pharmacology, School of Pharmacy, Fudan University, Shanghai 201203, China

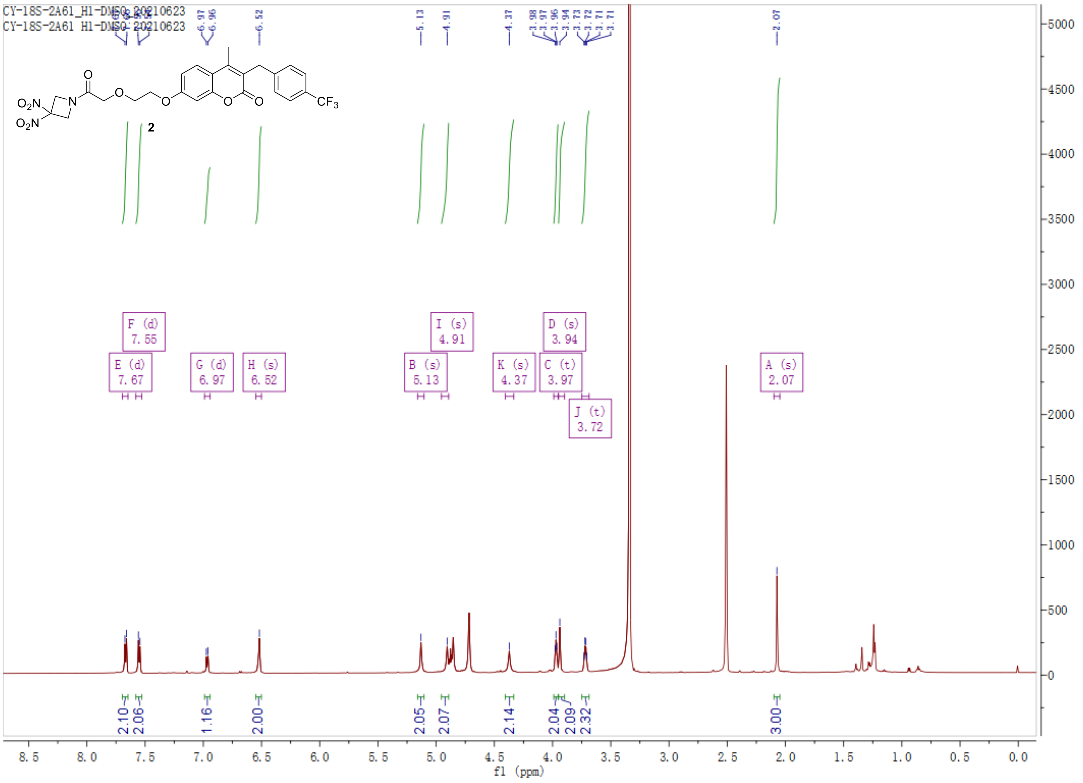

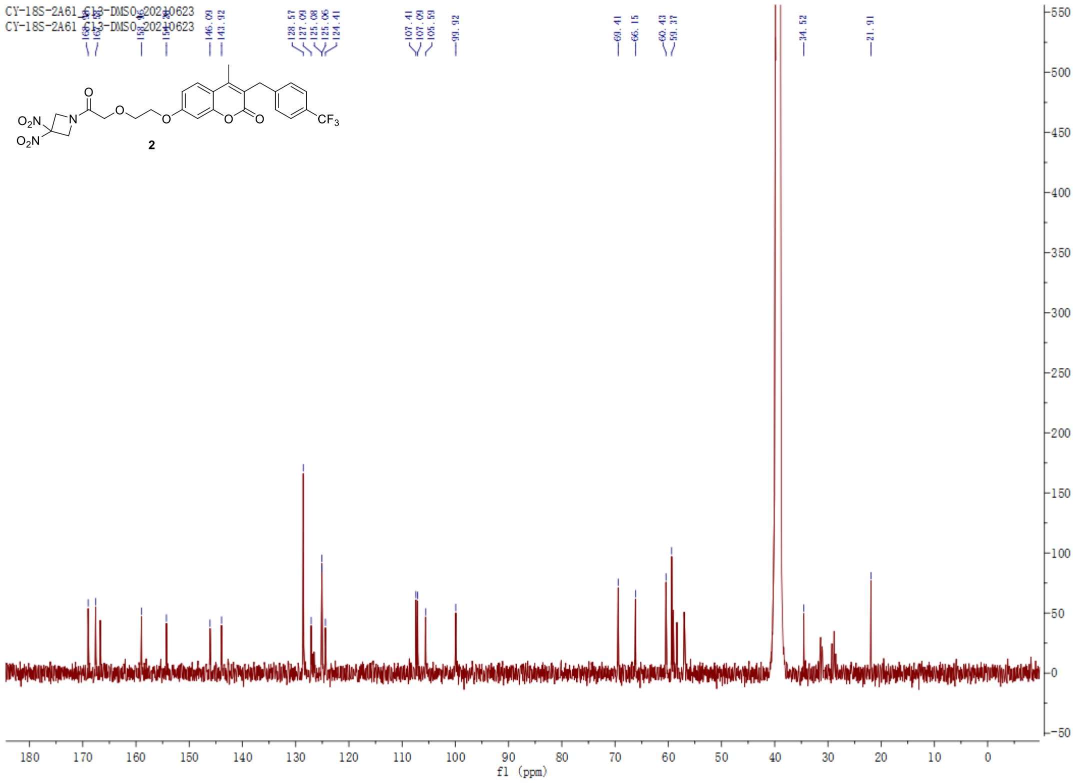

Figure S1. ^{1}H NMR (600 MHz, DMSO-d_6) and ^{13}C NMR (151 MHz, DMSO-d_6) spectrum of **2**.

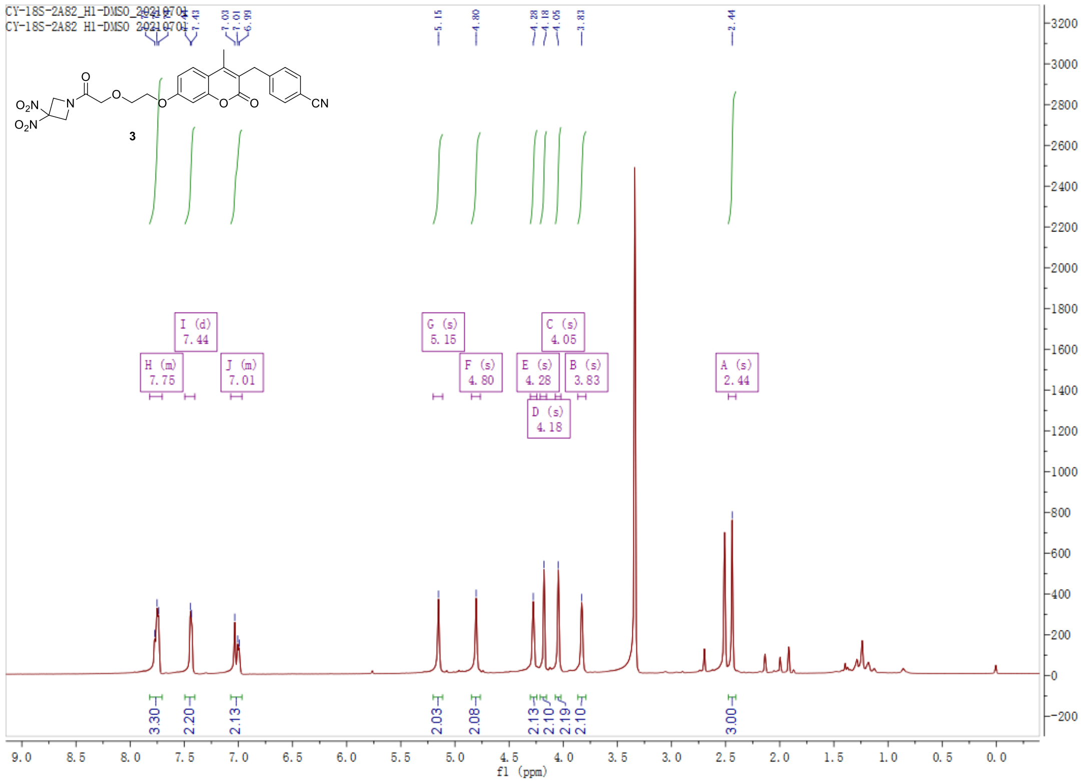

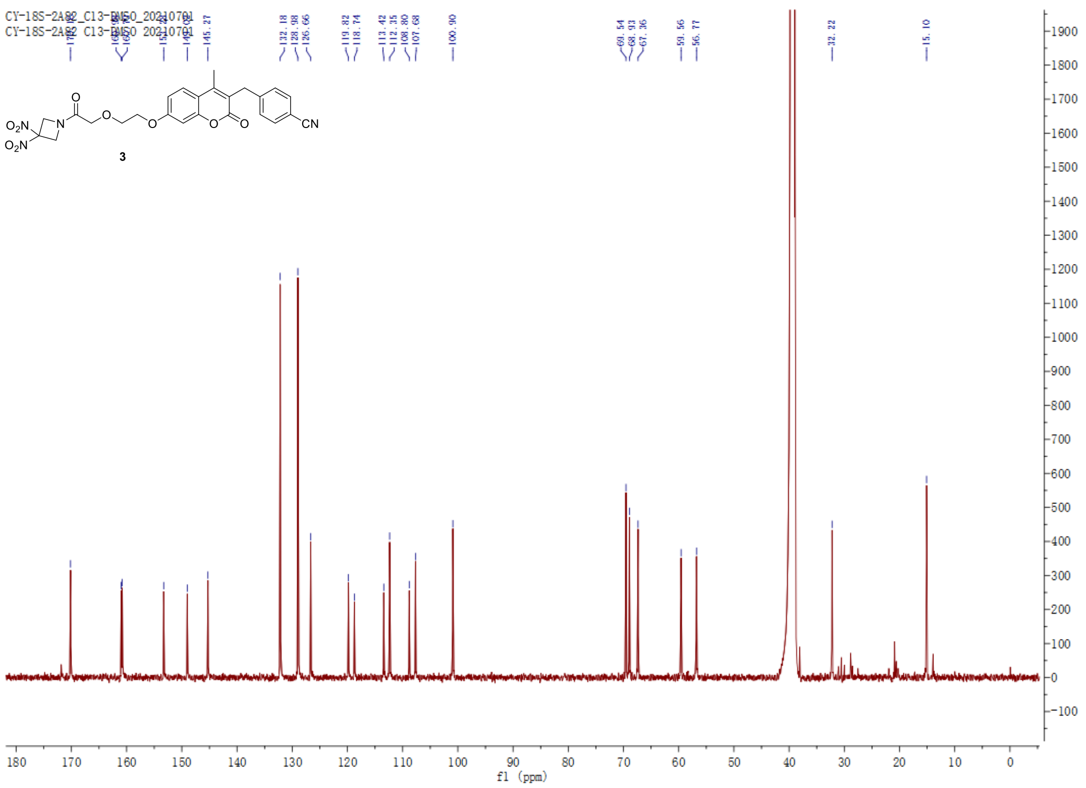

Figure S2. ^{1}H NMR (600 MHz, DMSO-d_6) and ^{13}C NMR (151 MHz, DMSO-d_6) spectrum of **3**.

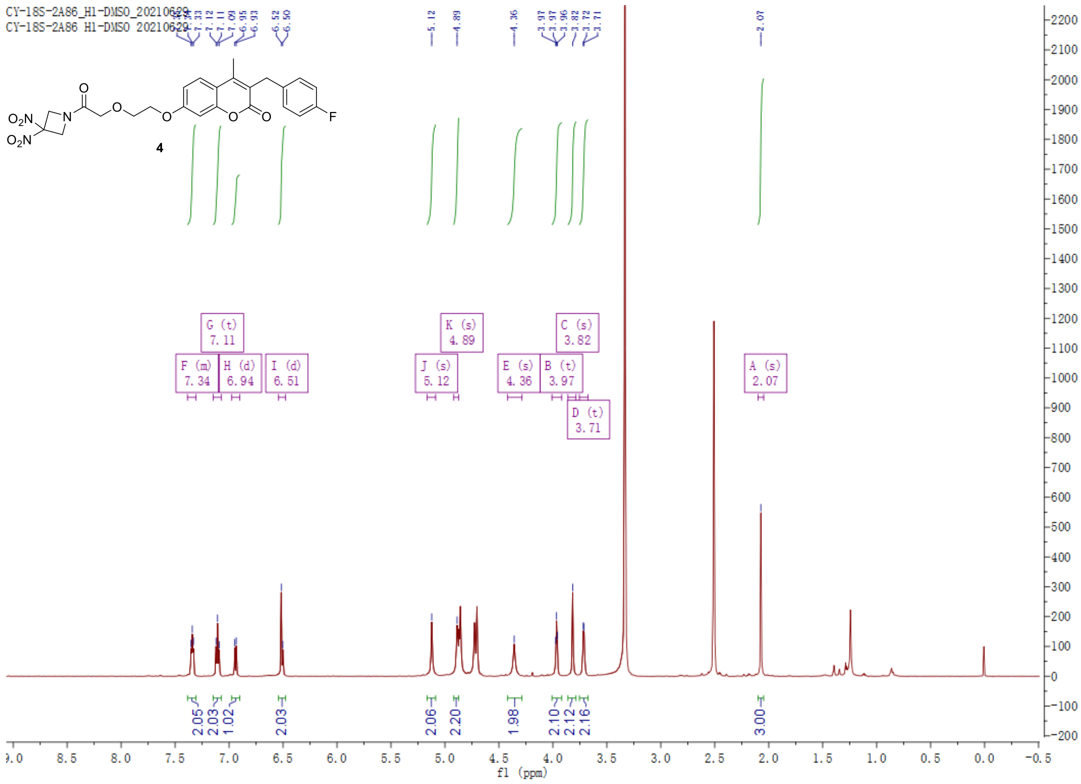

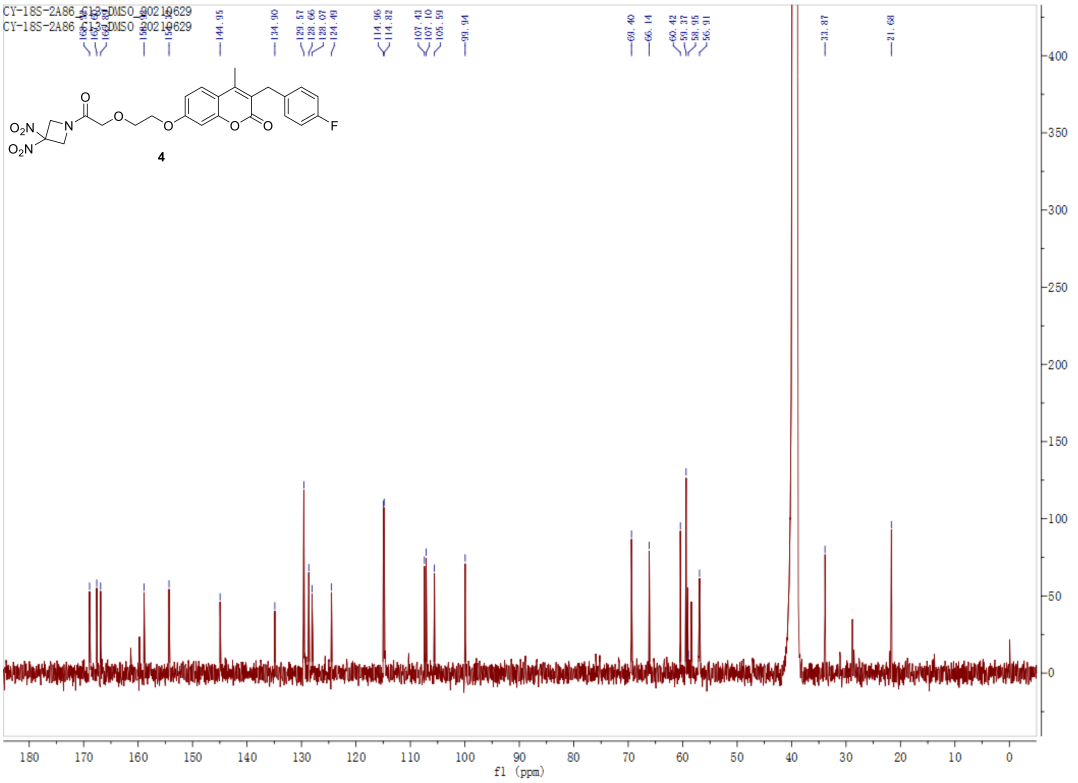

Figure S3. ^{1}H NMR (600 MHz, DMSO-d_6) and ^{13}C NMR (151 MHz, DMSO-d_6) spectrum of **4**.

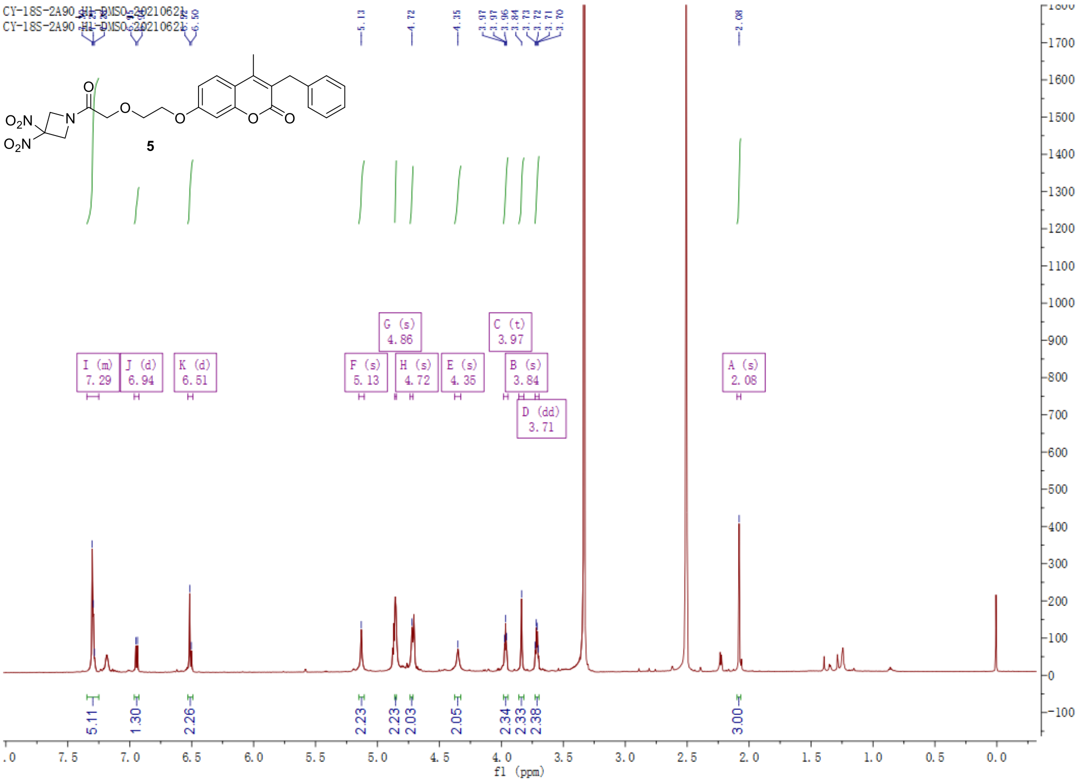

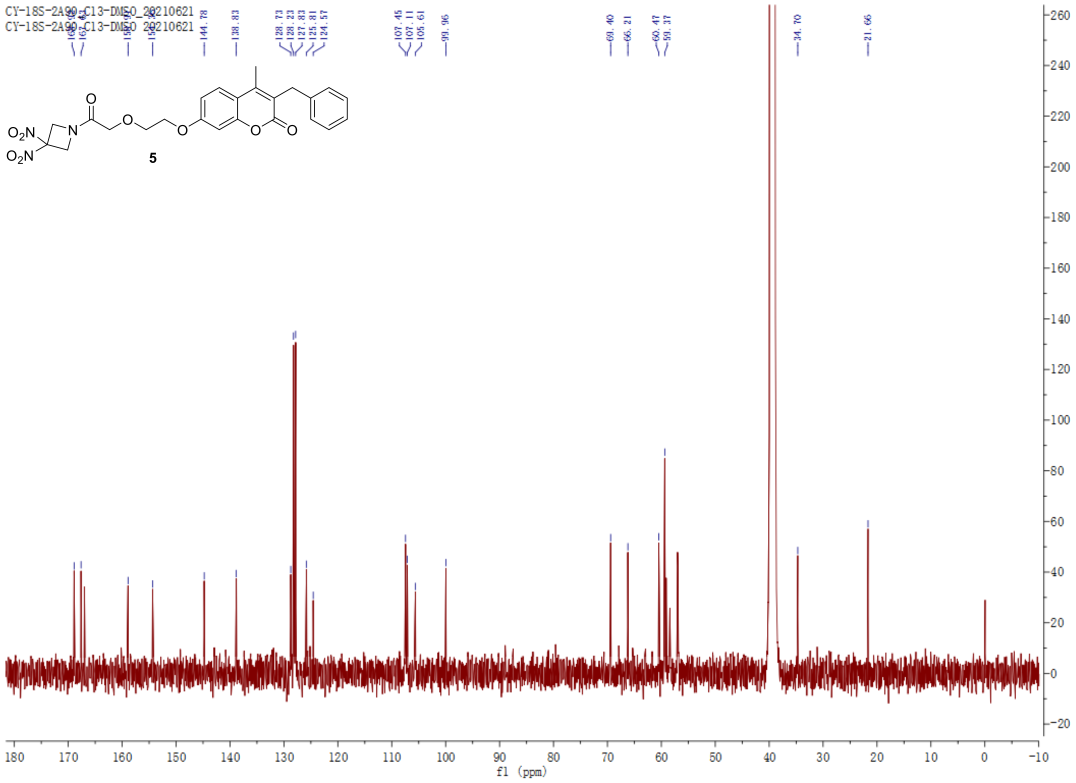

Figure S4. ^{1}H NMR (600 MHz, DMSO-d_6) and ^{13}C NMR (151 MHz, DMSO-d_6) spectrum of **5**.

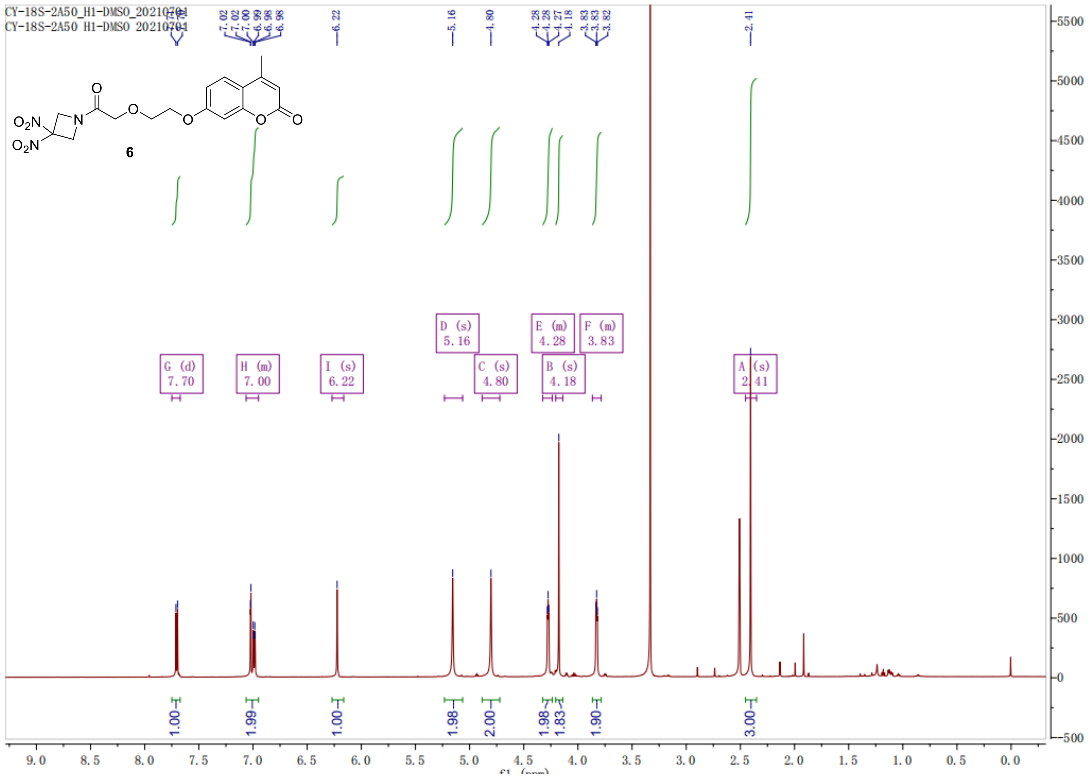

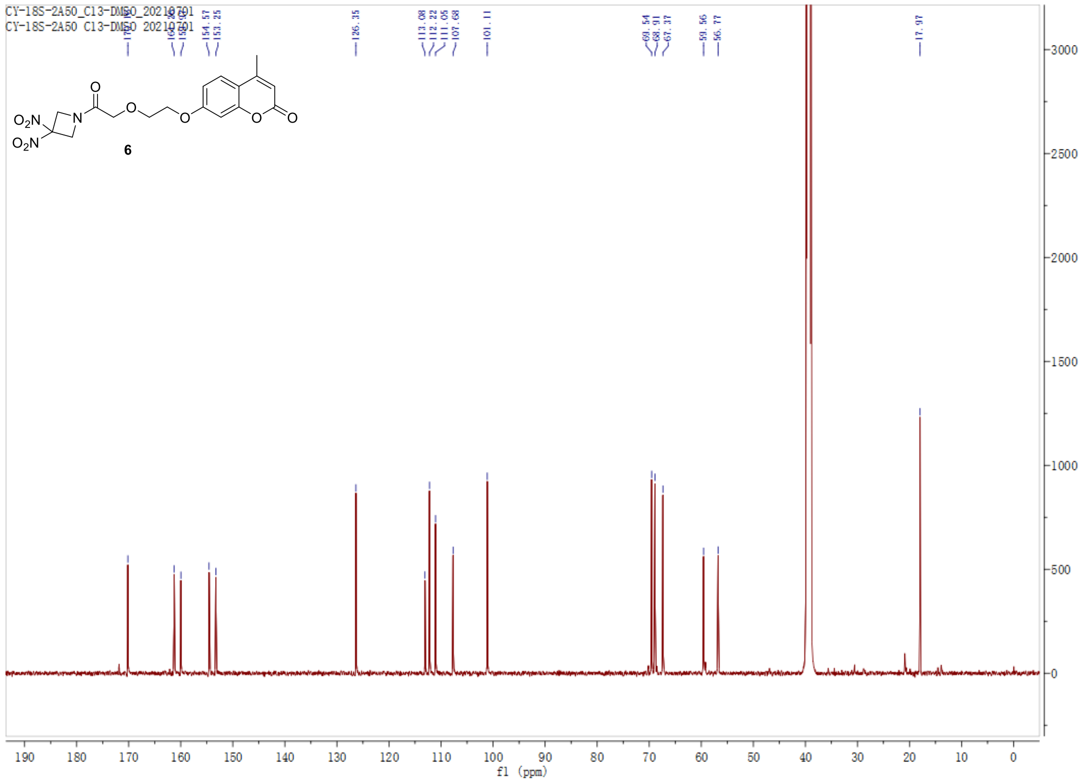

Figure S5. ^{1}H NMR (600 MHz, DMSO-d_6) and ^{13}C NMR (151 MHz, DMSO-d_6) spectrum of **6**.

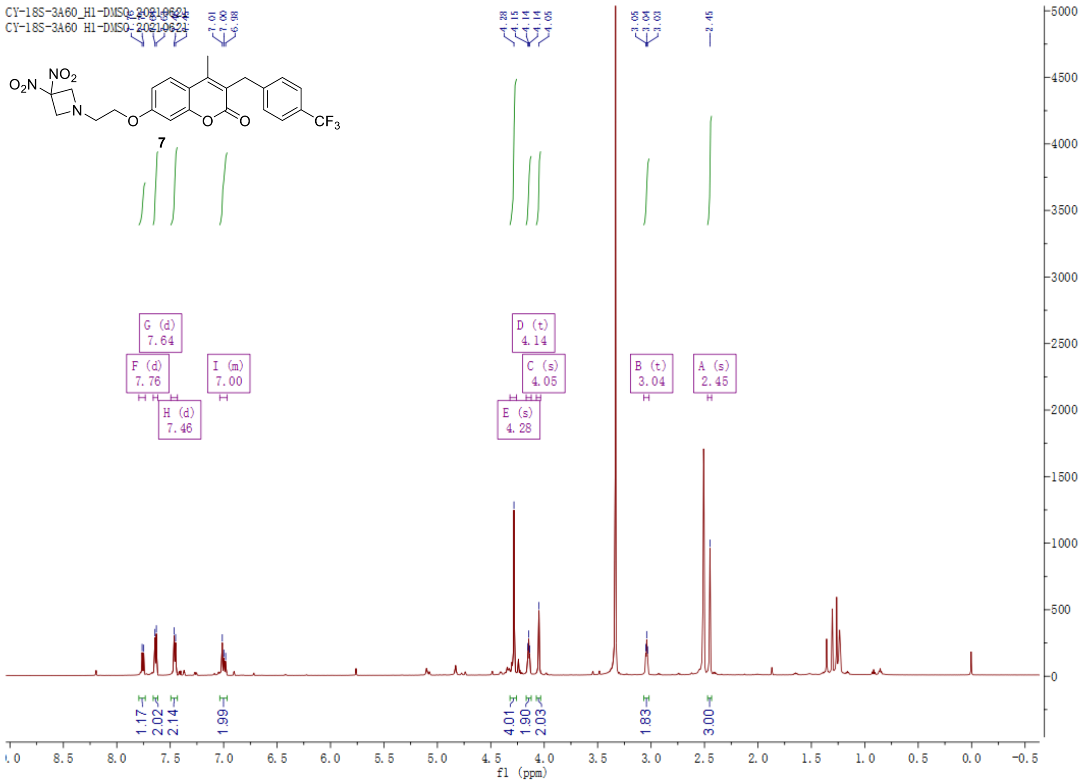

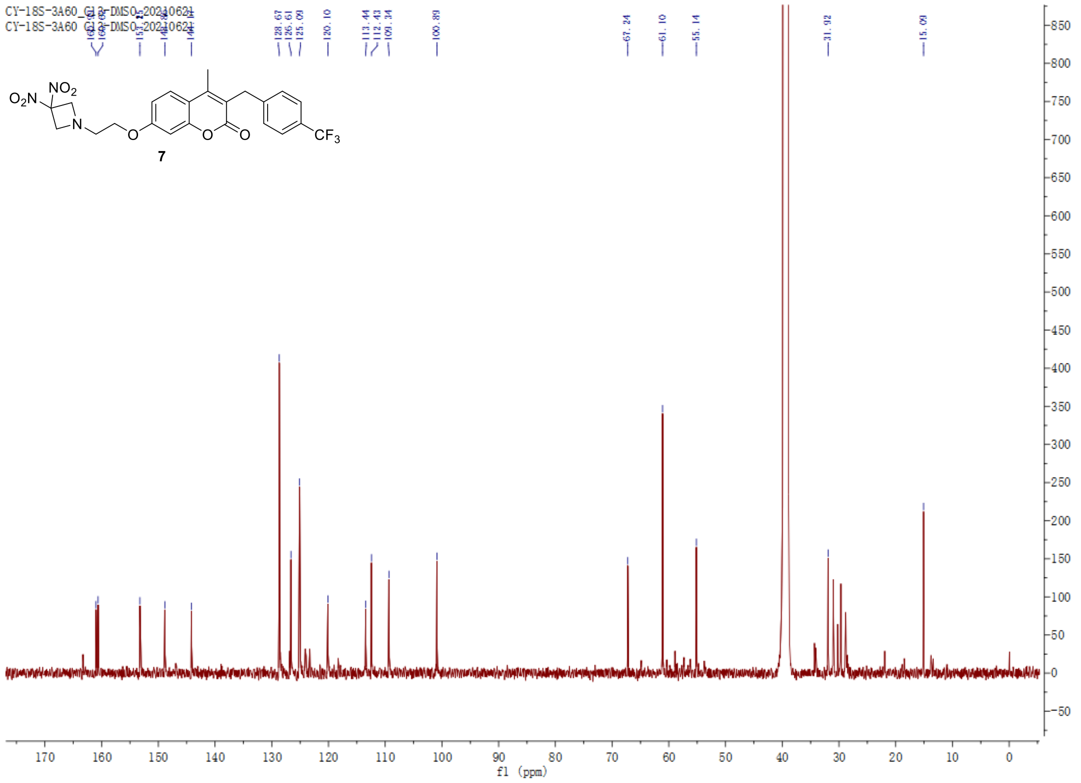

Figure S6. ^{1}H NMR (600 MHz, DMSO-d_6) and ^{13}C NMR (151 MHz, DMSO-d_6) spectrum of **7**.

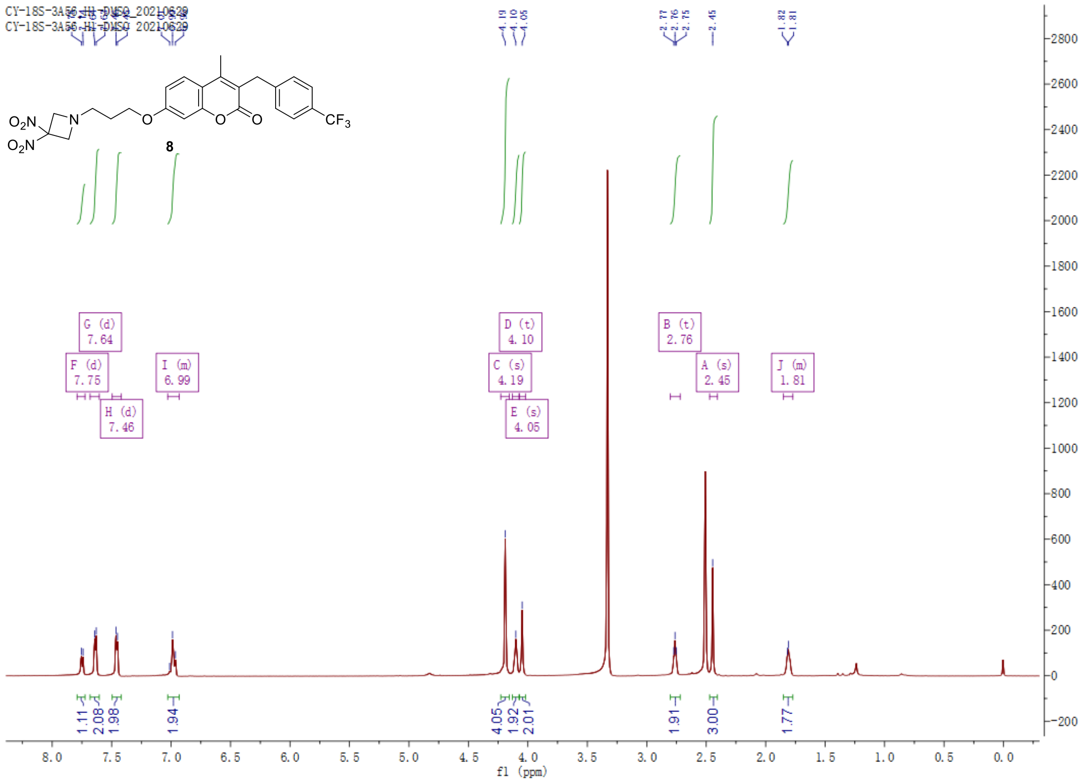

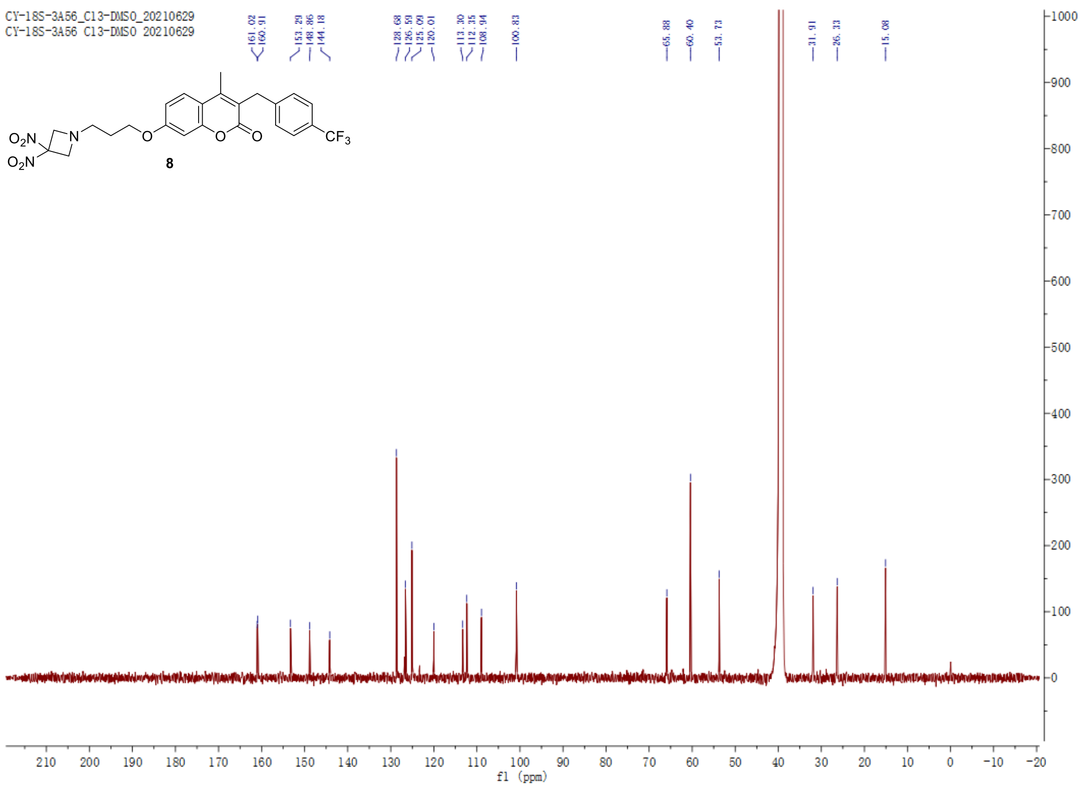

Figure S7. ^{1}H NMR (600 MHz, DMSO-d_6) and ^{13}C NMR (151 MHz, DMSO-d_6) spectrum of **8**.

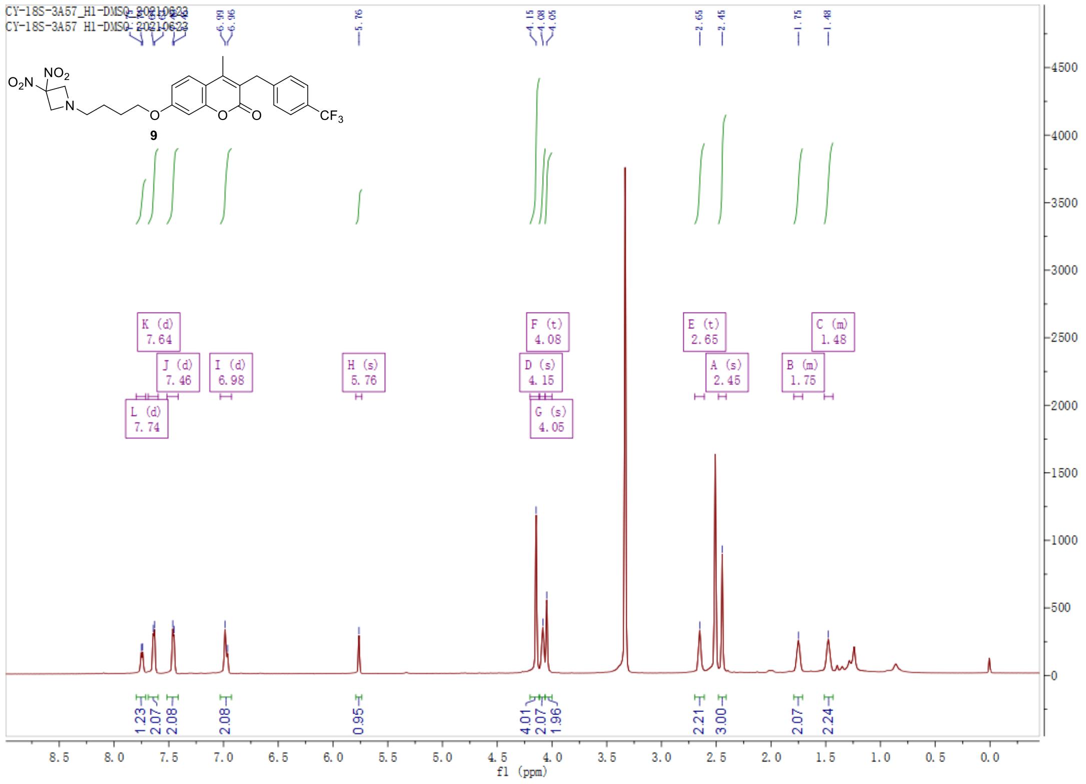

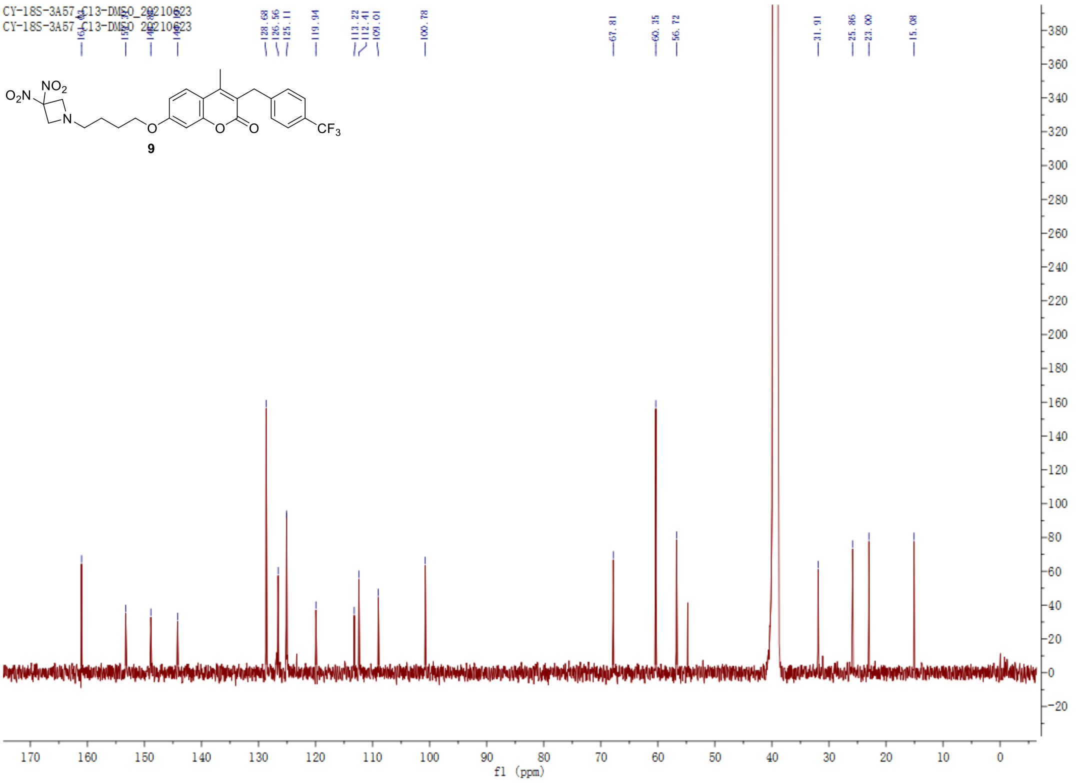

Figure S8. ^{1}H NMR (600 MHz, DMSO-d_6) and ^{13}C NMR (151 MHz, DMSO-d_6) spectrum of **9**.

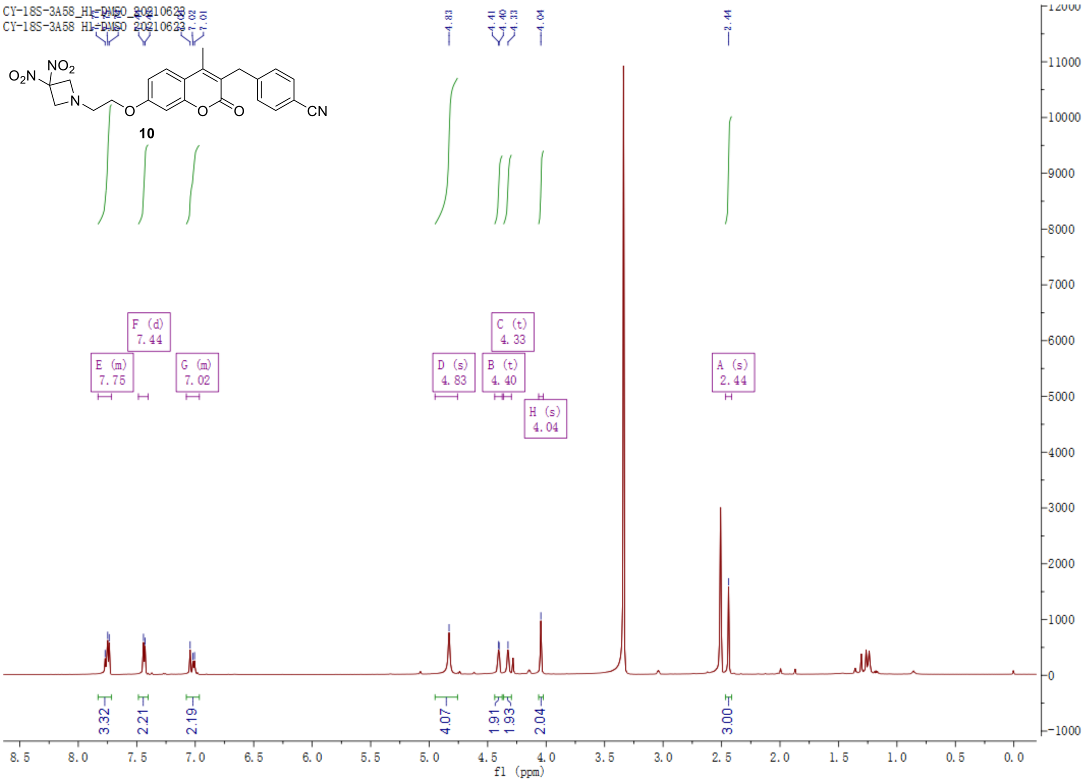

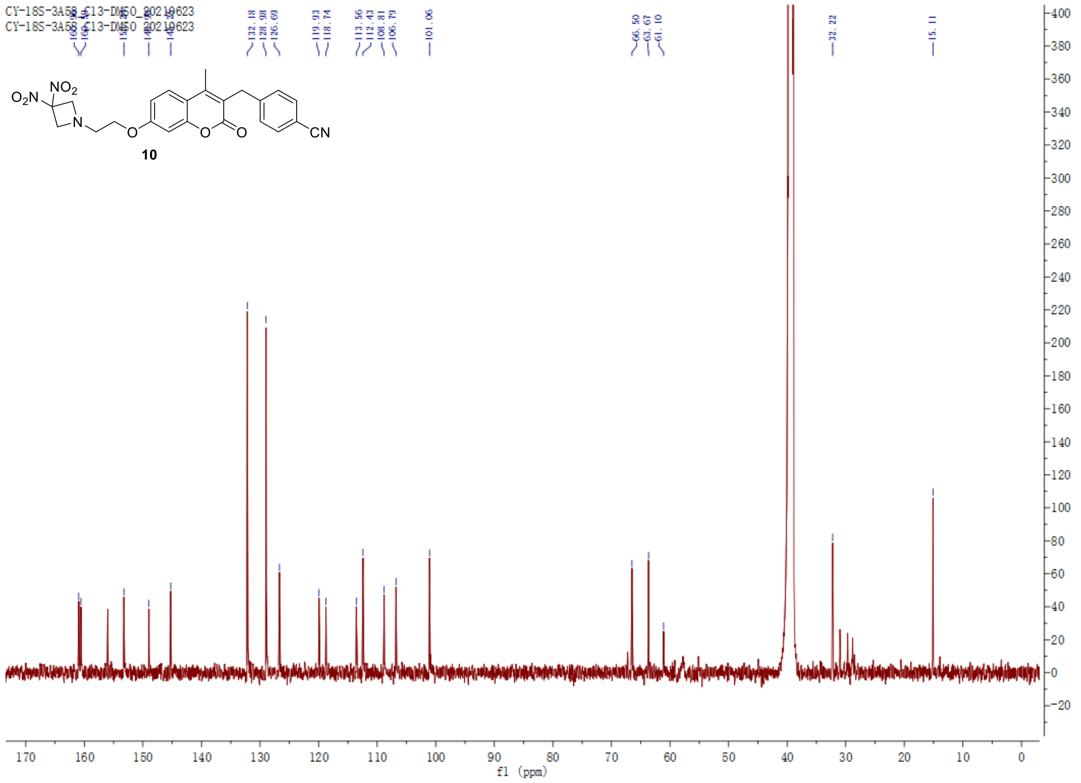

Figure S9. ^{1}H NMR (600 MHz, DMSO-d_6) and ^{13}C NMR (151 MHz, DMSO-d_6) spectrum of **10**.

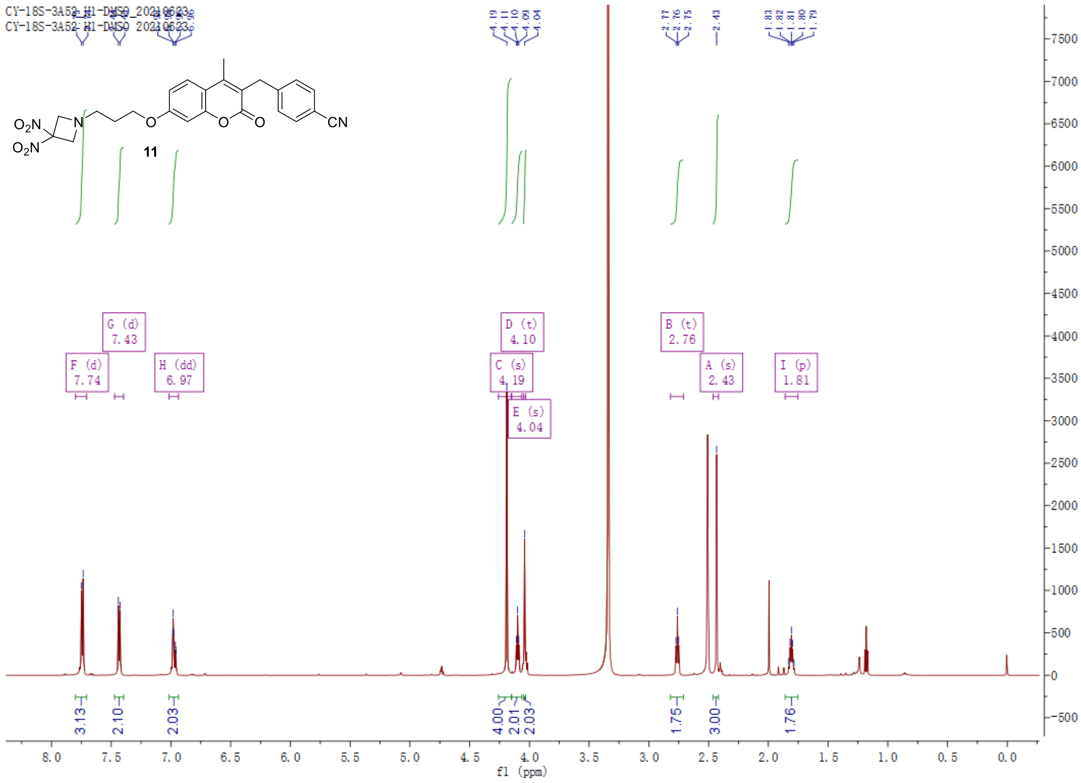

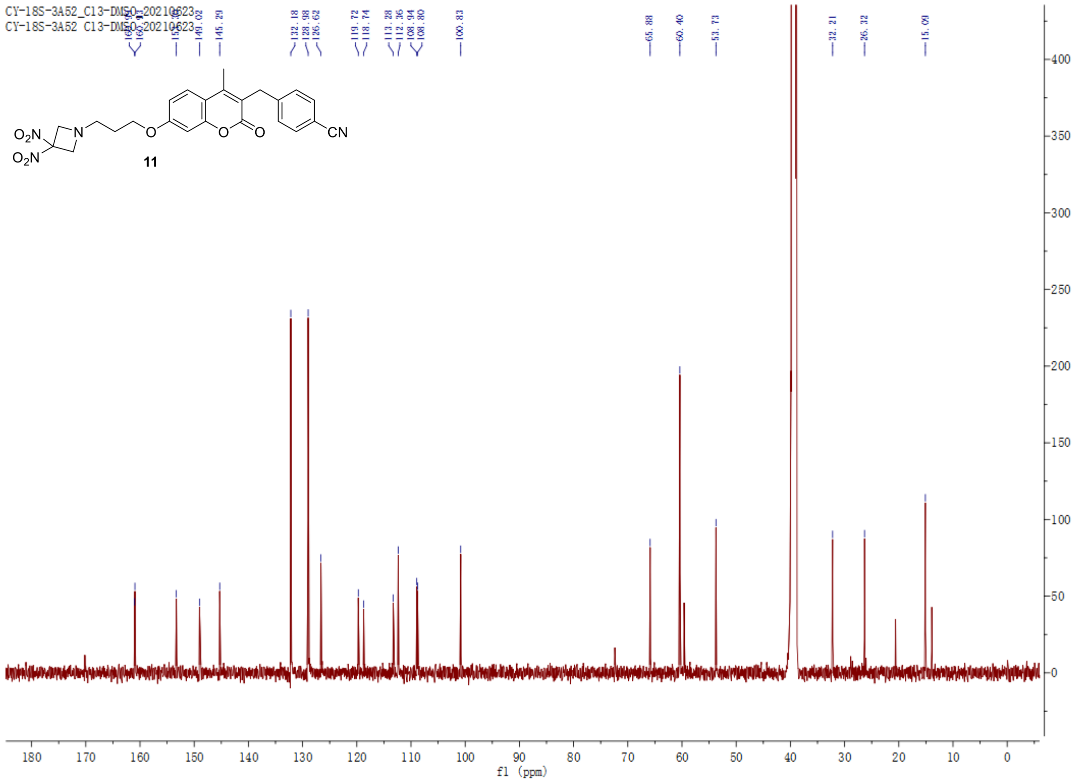

Figure S10. ^{1}H NMR (600 MHz, DMSO-d_6) and ^{13}C NMR (151 MHz, DMSO-d_6) spectrum of **11**.

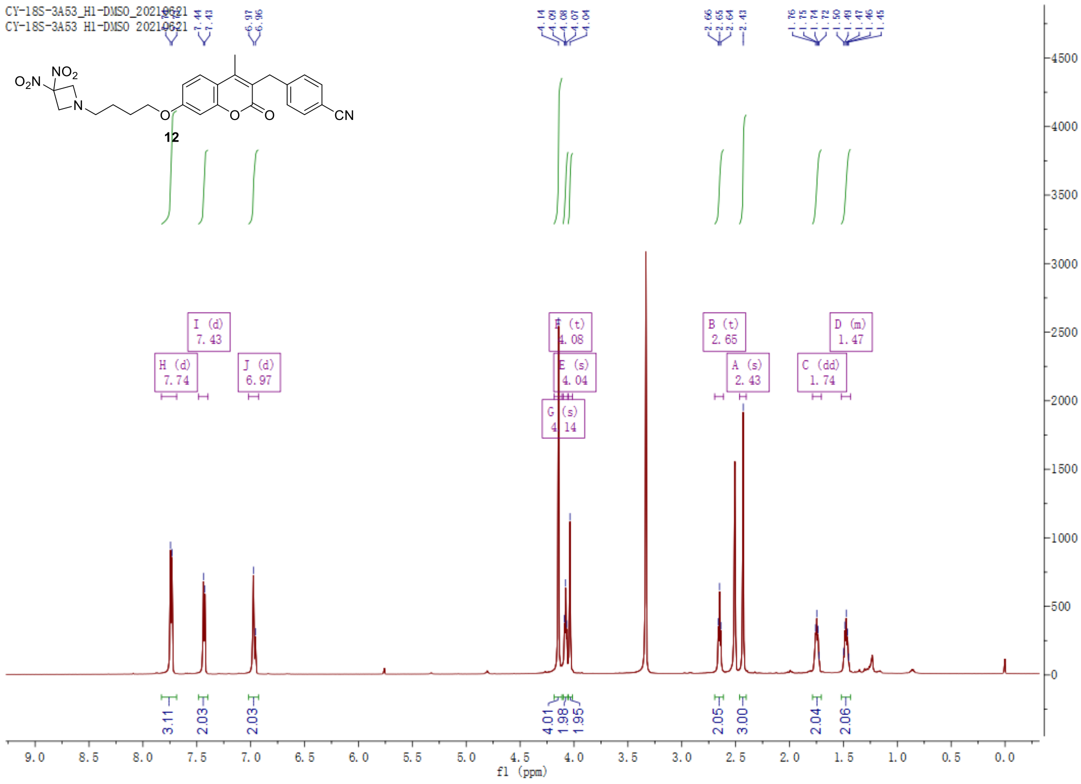

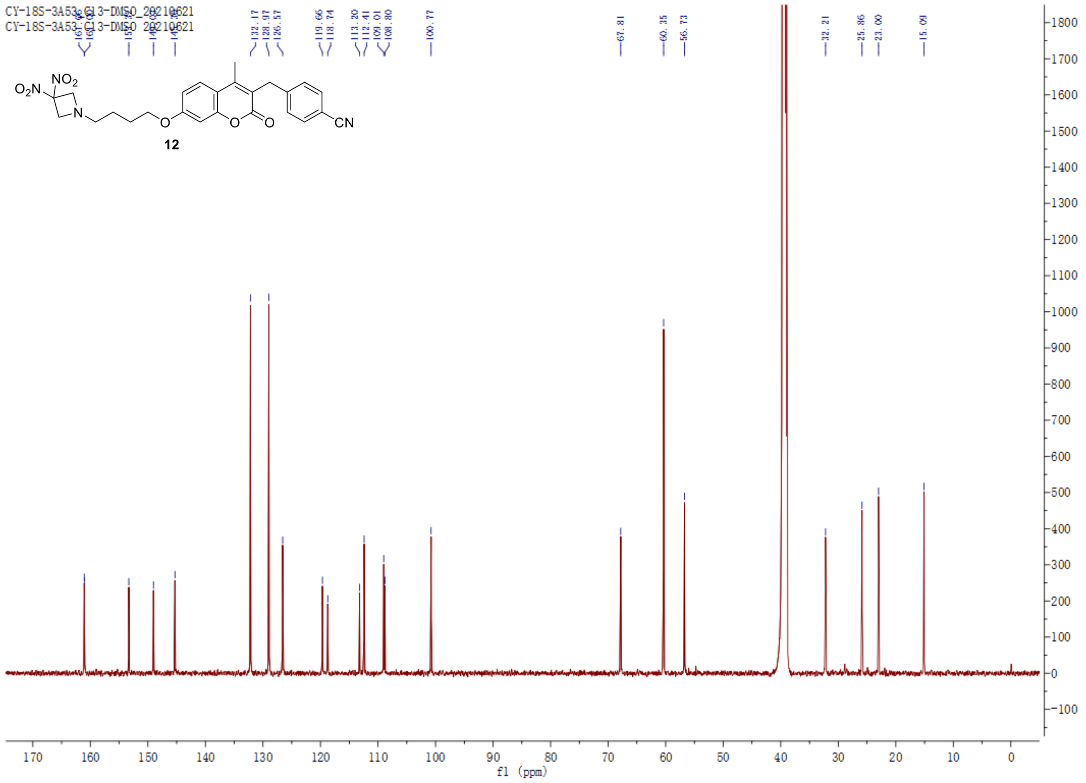

Figure S11. ^{1}H NMR (600 MHz, DMSO-d_6) and ^{13}C NMR (151 MHz, DMSO-d_6) spectrum of 1**2**.

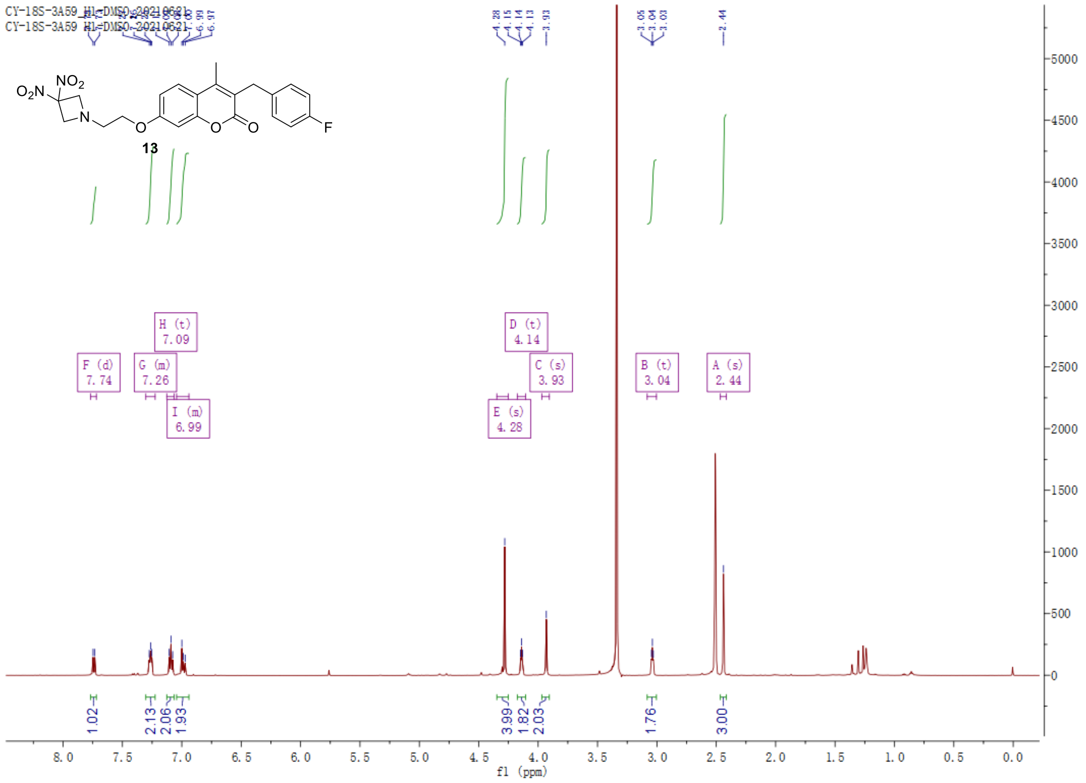

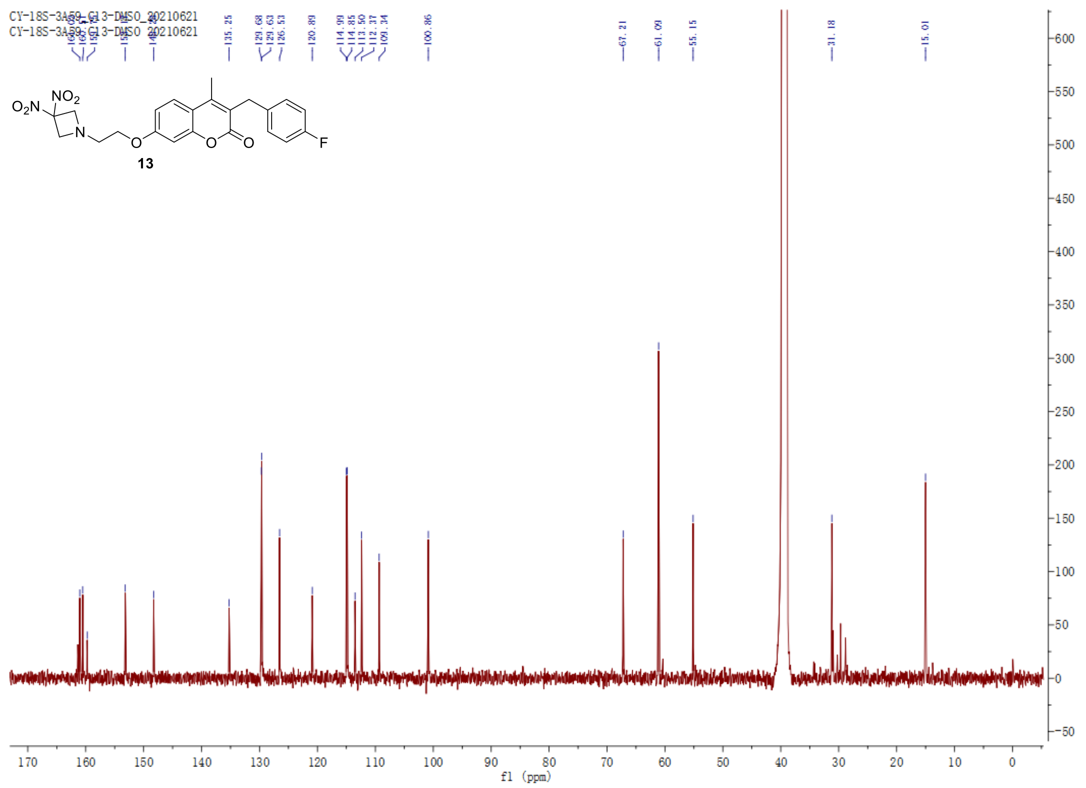

Figure S12. ^{1}H NMR (600 MHz, DMSO-d_6) and ^{13}C NMR (151 MHz, DMSO-d_6) spectrum of **13**.

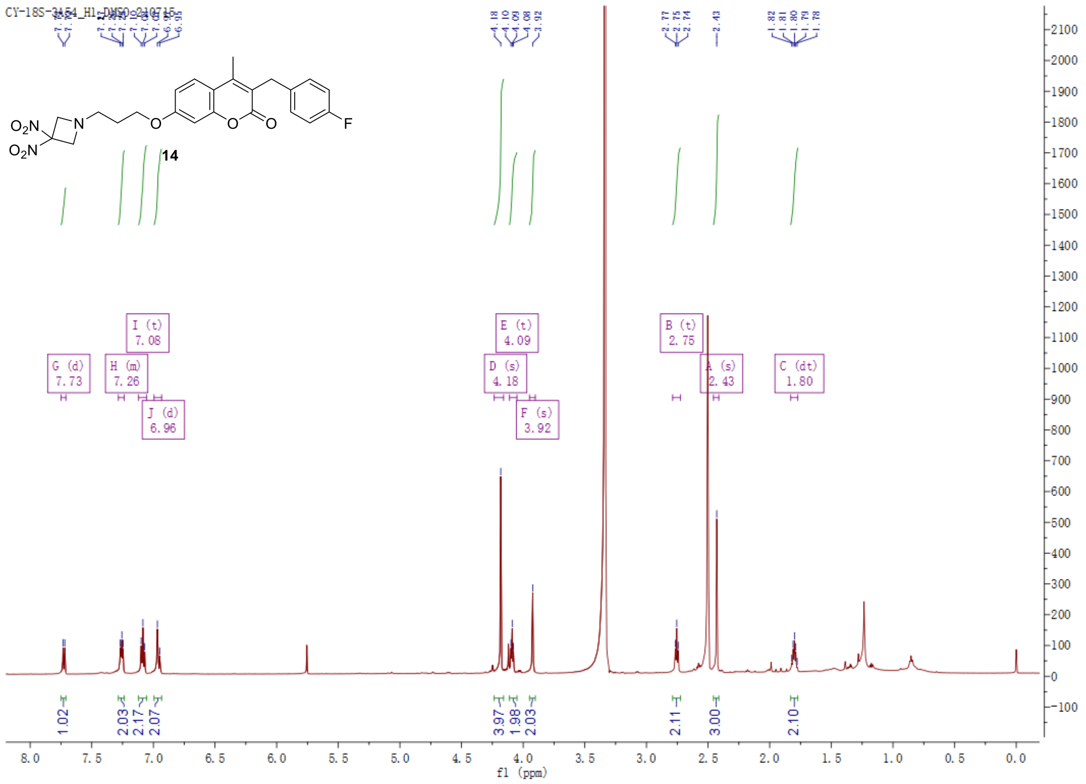

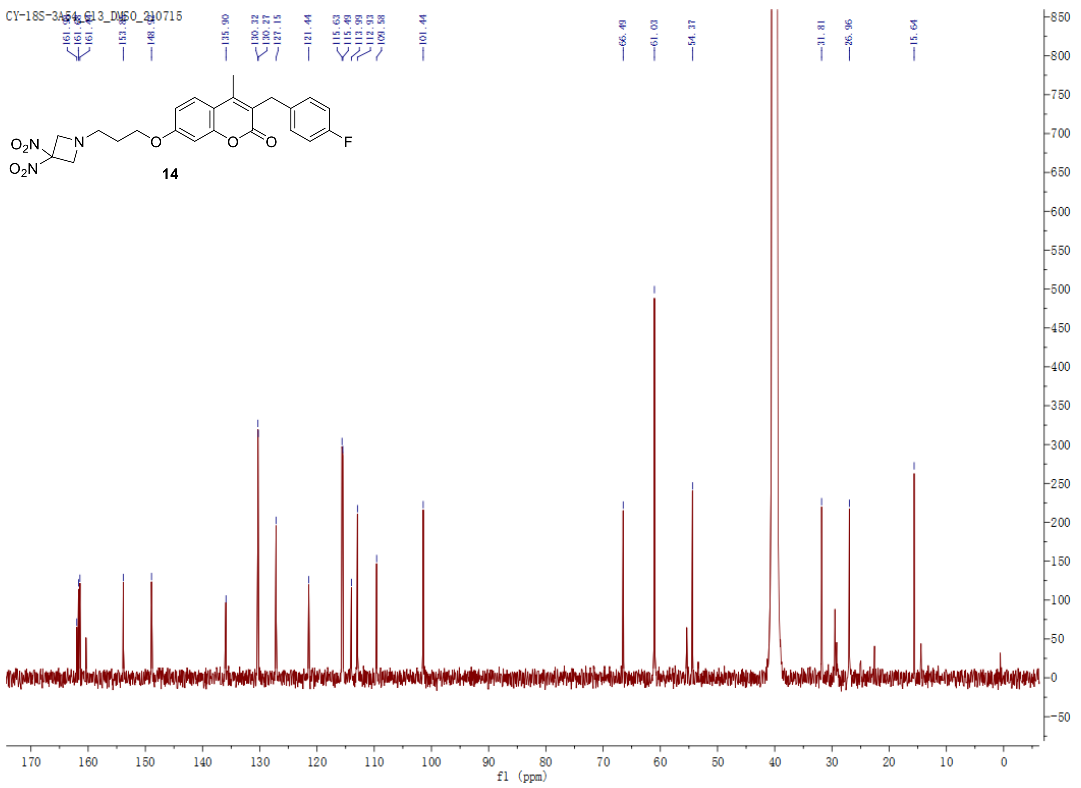

Figure S13. ^{1}H NMR (600 MHz, DMSO-d_6) and ^{13}C NMR (151 MHz, DMSO-d_6) spectrum of **14**.

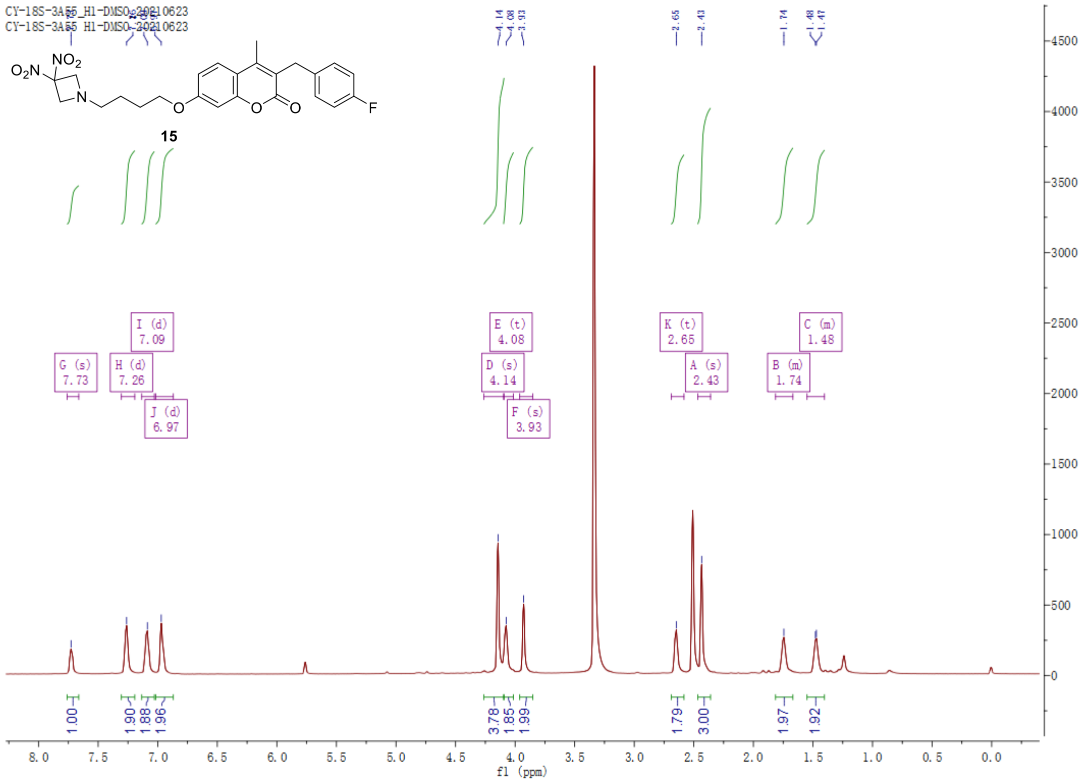

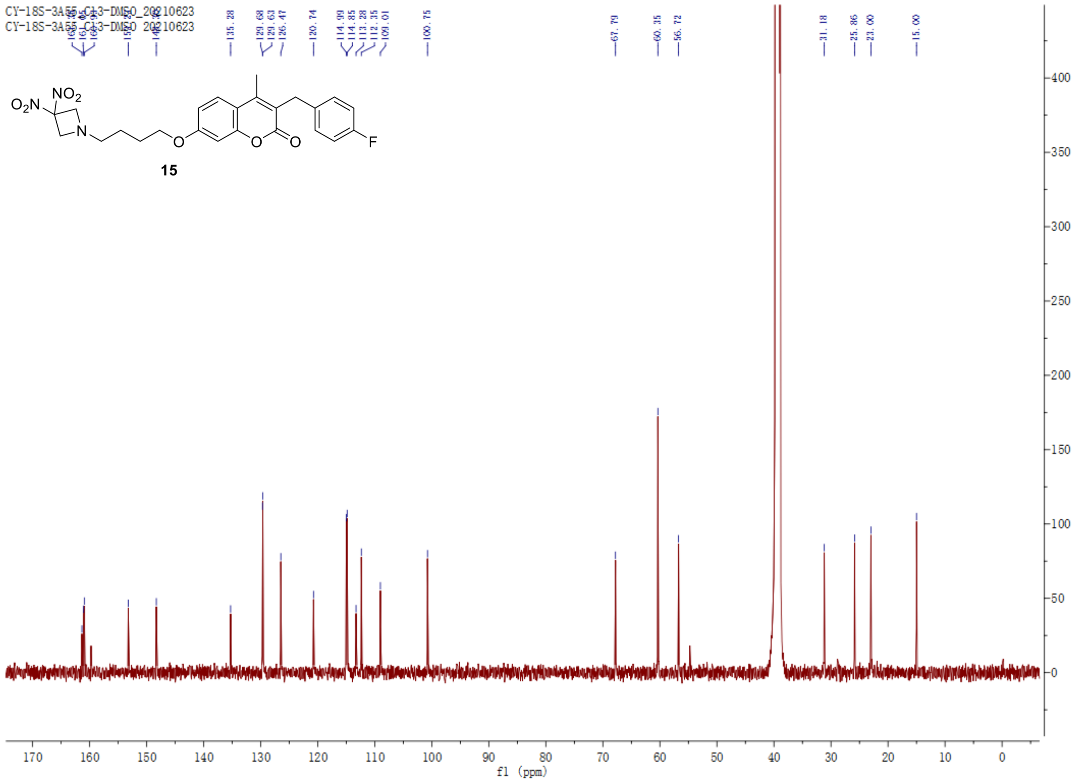

Figure S14. ^{1}H NMR (600 MHz, DMSO-d_6) and ^{13}C NMR (151 MHz, DMSO-d_6) spectrum of **15**.

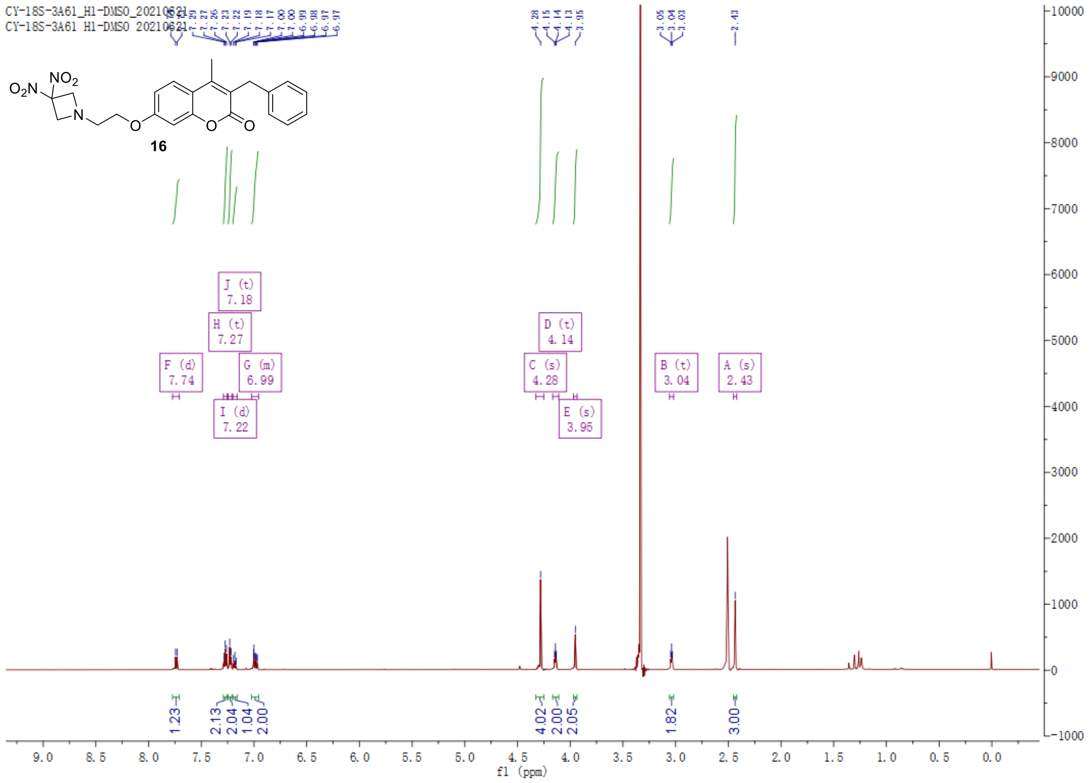

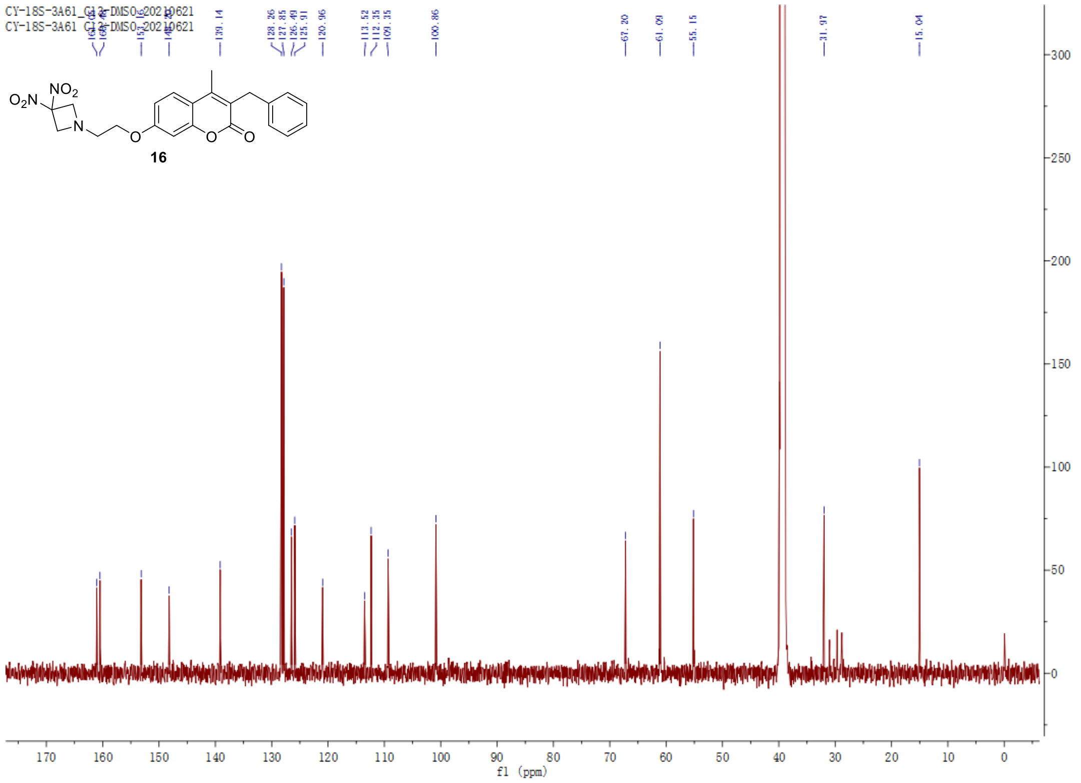

Figure S15. ^{1}H NMR (600 MHz, DMSO-d_6) and ^{13}C NMR (151 MHz, DMSO-d_6) spectrum of **16**.

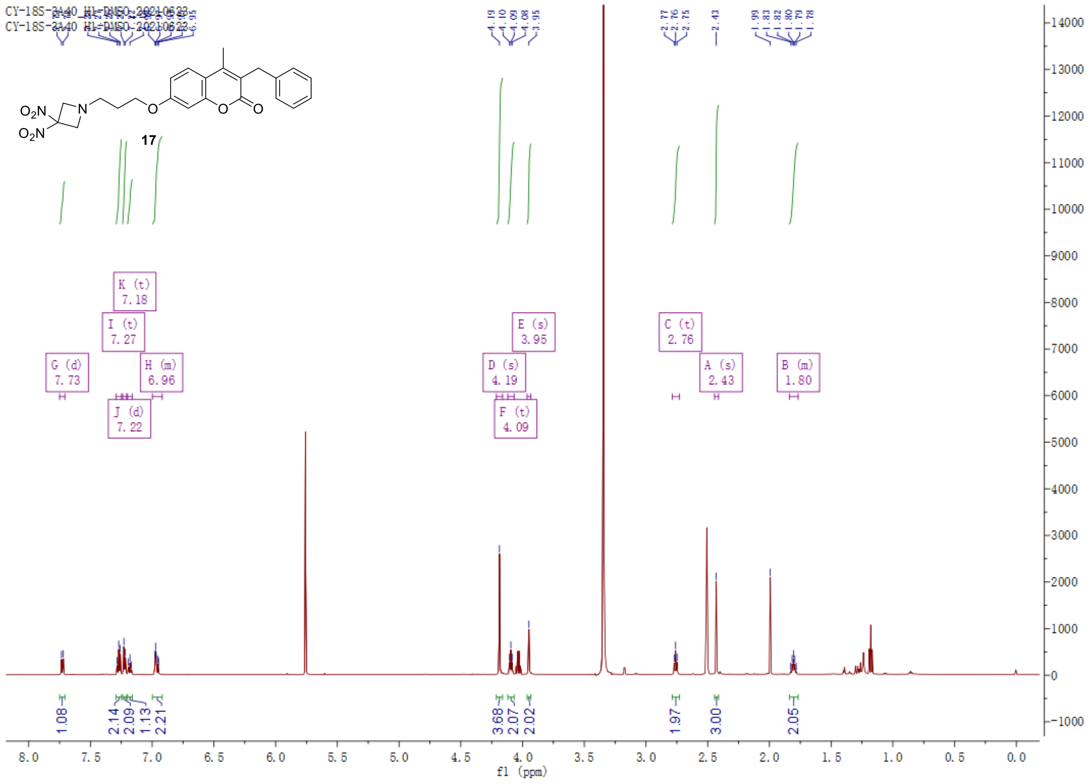

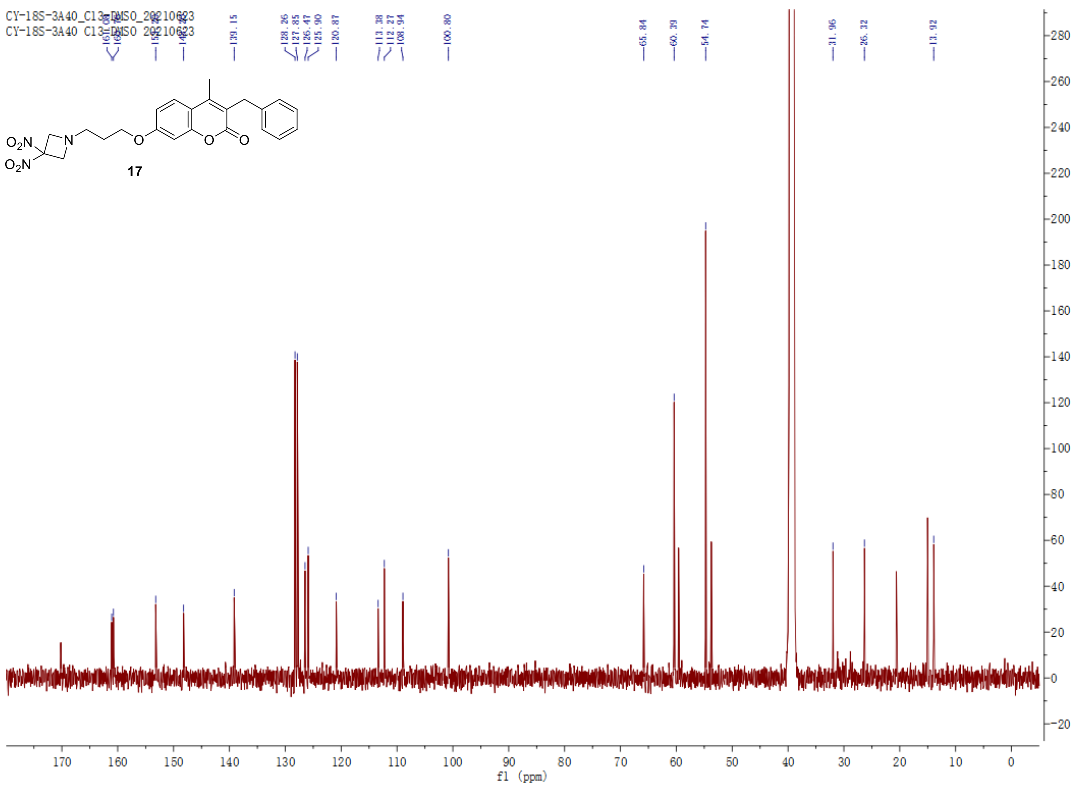

Figure S16. ^{1}H NMR (600 MHz, DMSO-d_6) and ^{13}C NMR (151 MHz, DMSO-d_6) spectrum of **17**.

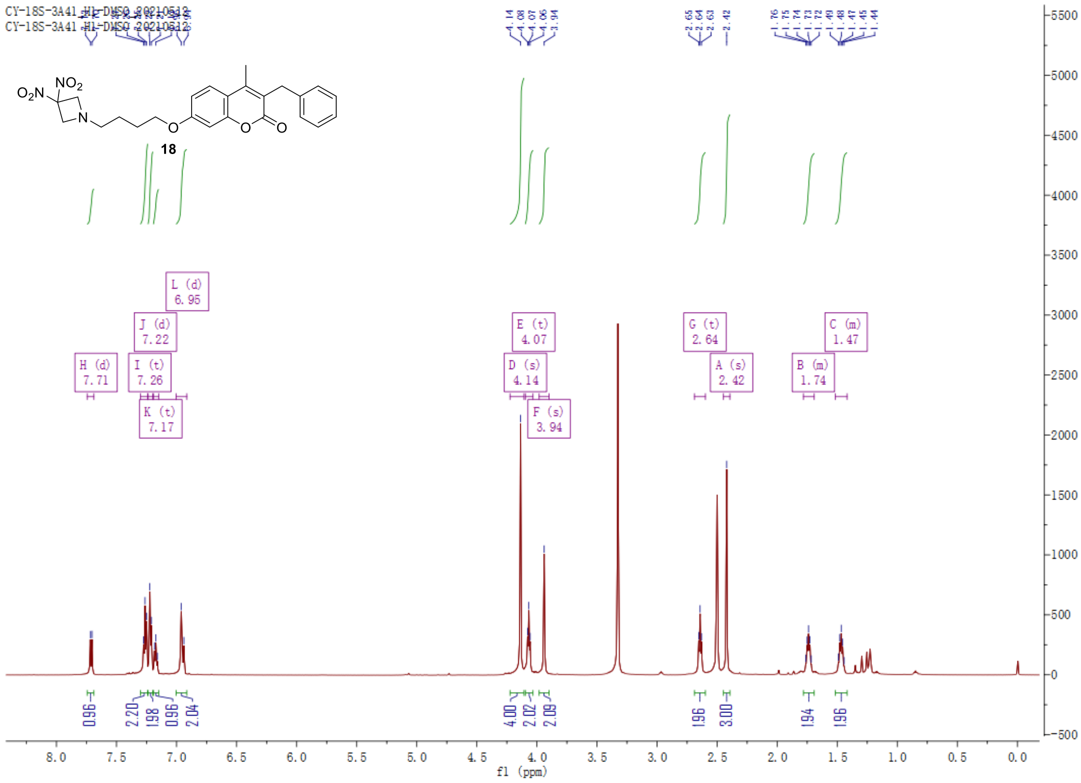

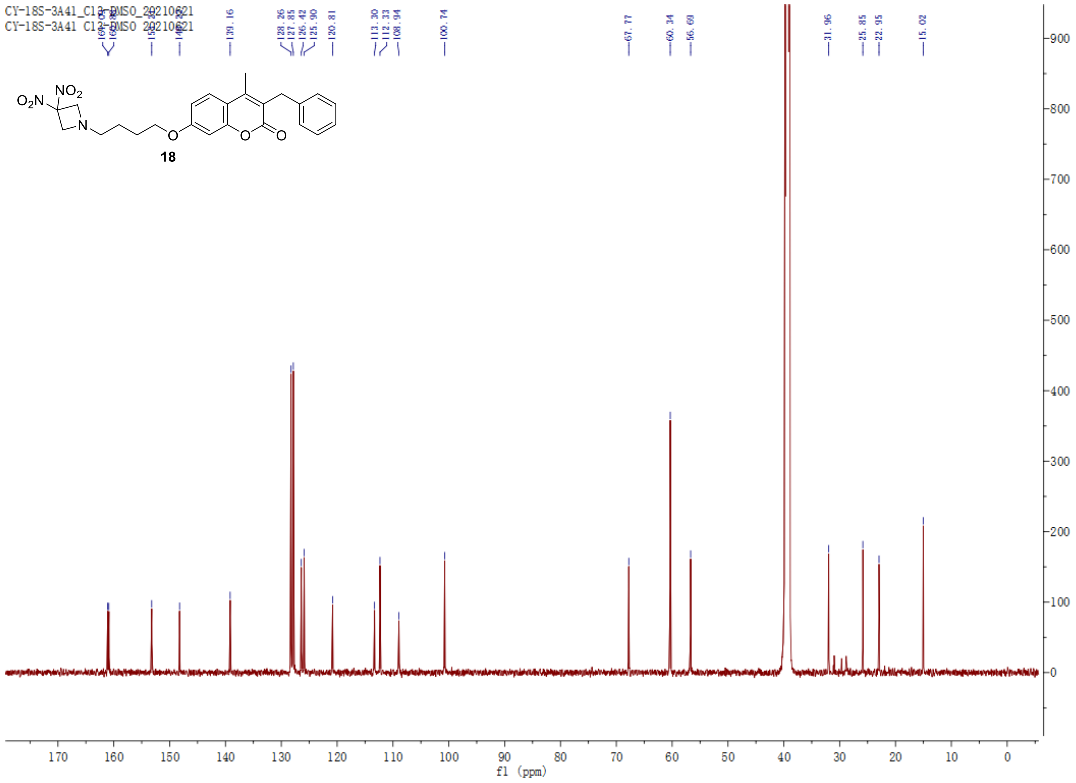

Figure S17. ^{1}H NMR (600 MHz, DMSO-d_6) and ^{13}C NMR (151 MHz, DMSO-d_6) spectrum of **18**.

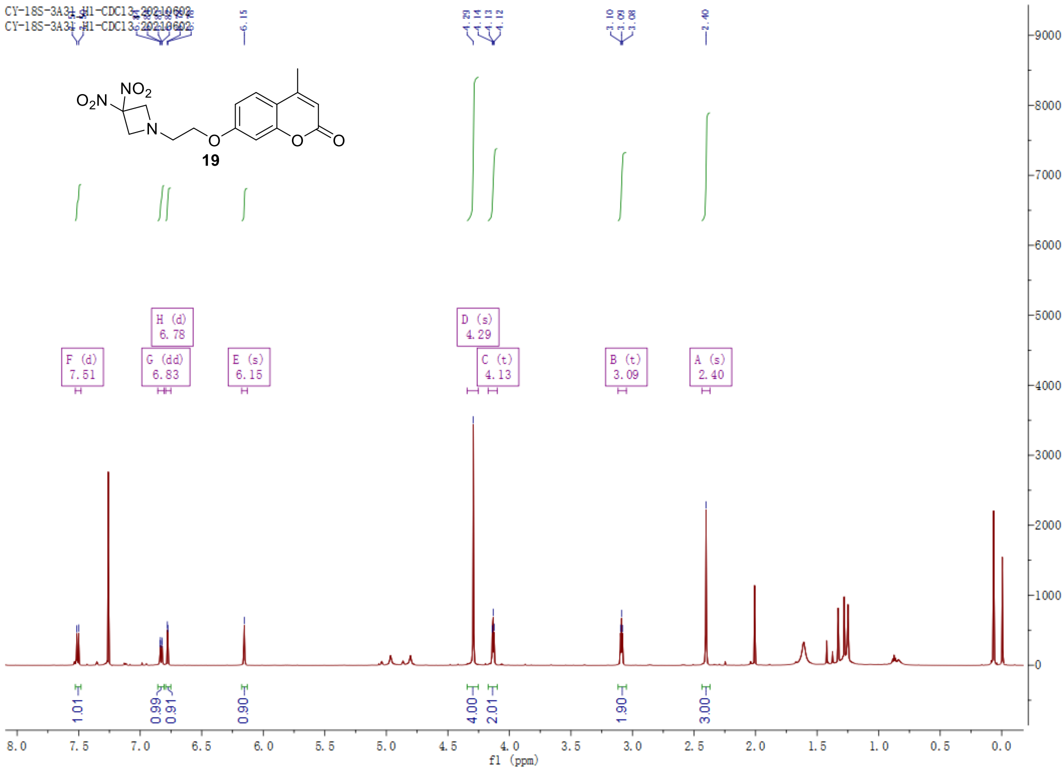

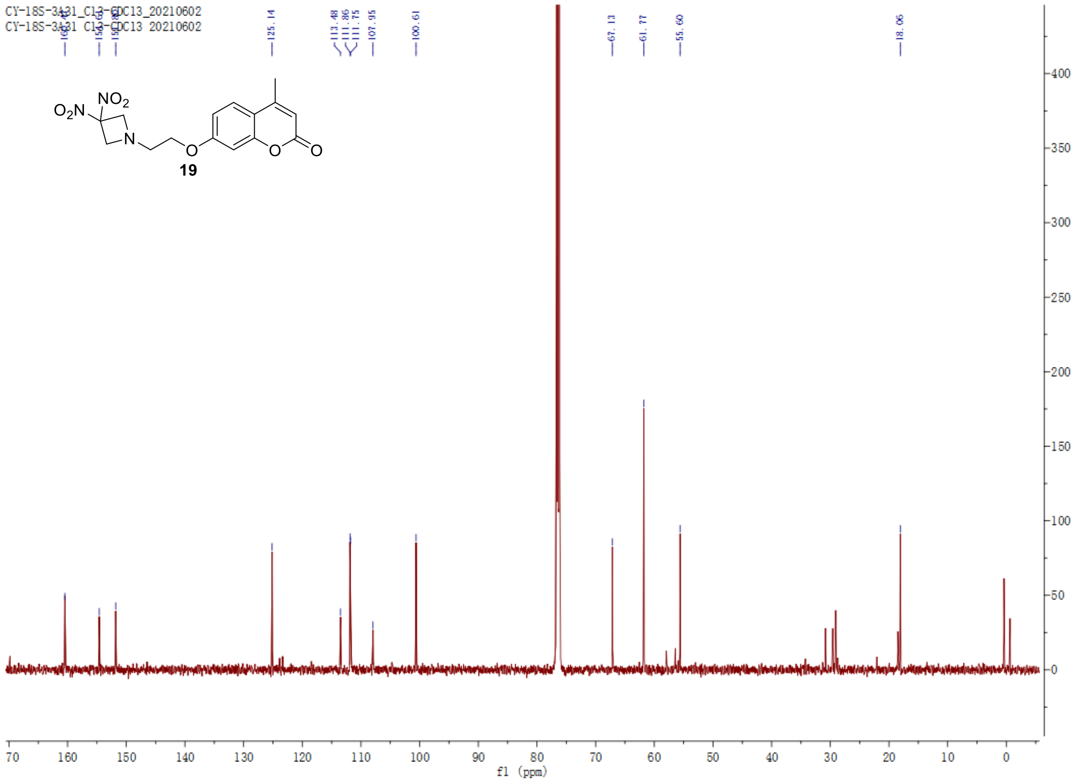

Figure S18. ^{1}H NMR (600 MHz, $CDCl_3$) and ^{13}C NMR (151 MHz, $CDCl_3$) spectrum of **19**.

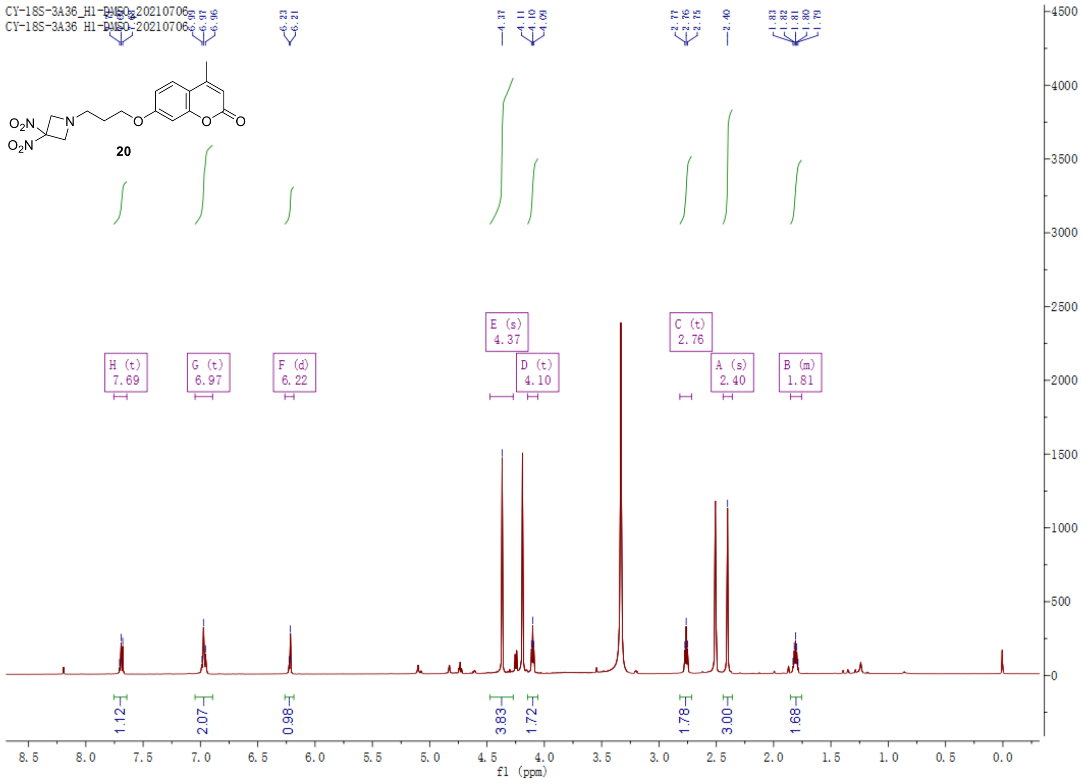

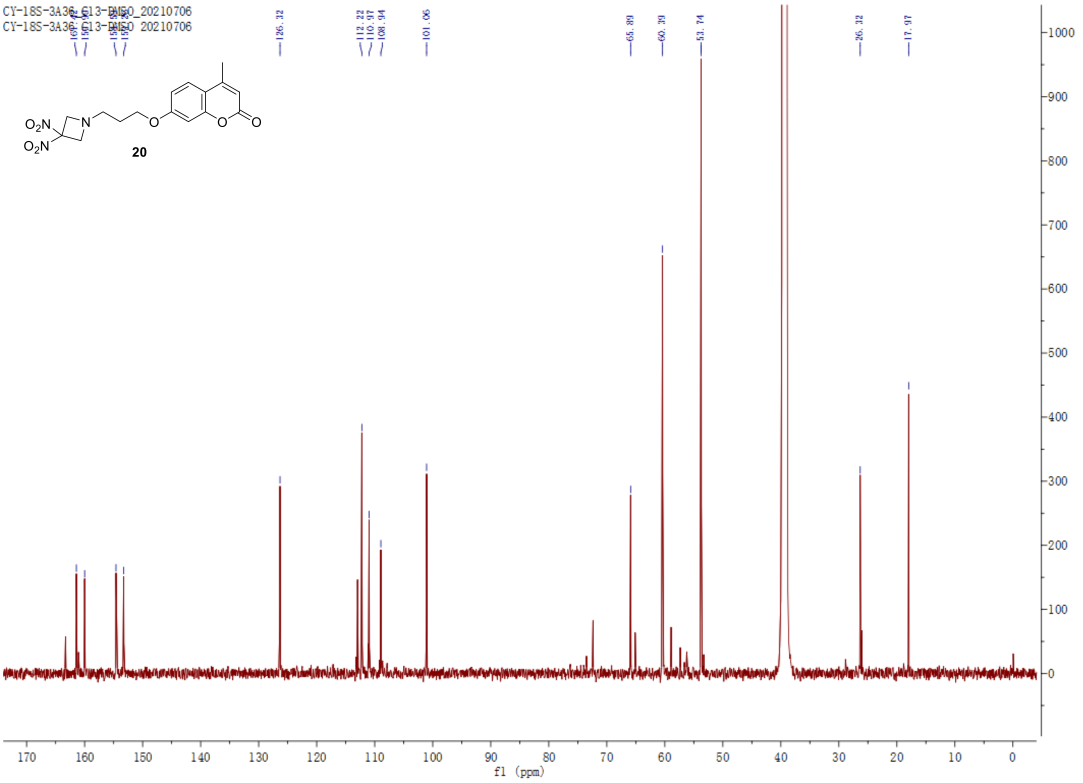

Figure S19. ^{1}H NMR (600 MHz, DMSO-d_6) and ^{13}C NMR (151 MHz, DMSO-d_6) spectrum of **20**.

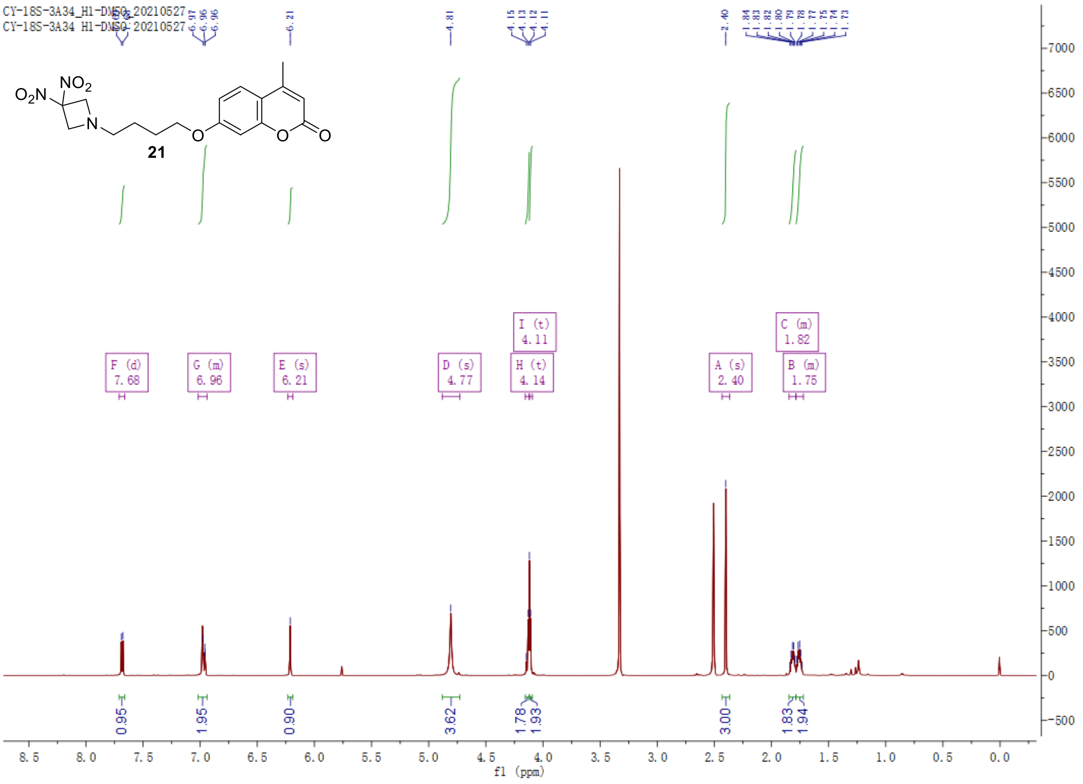

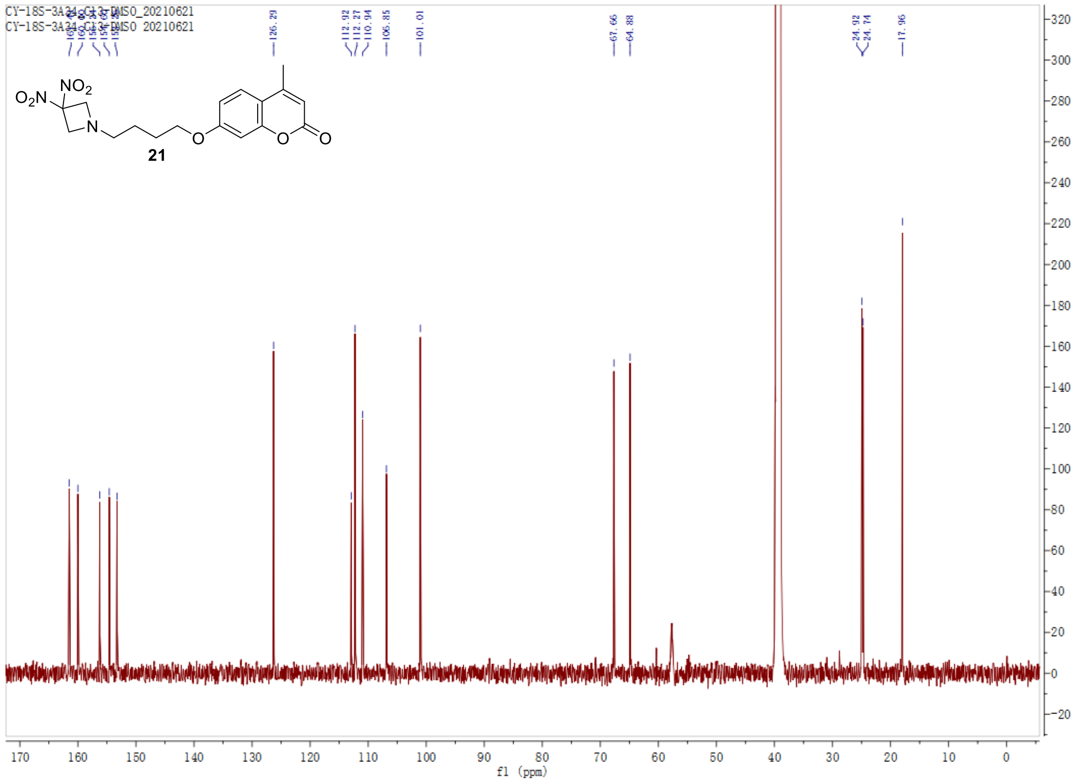

Figure S20. ^{1}H NMR (600 MHz, DMSO-d_6) and ^{13}C NMR (151 MHz, DMSO-d_6) spectrum of **21**.
